# Closer Peptide Repertoire Similarity of HLA-B∗14:03 and HLA-B∗27:05 Sheds Light on Ankylosing Spondylitis Susceptibility

**DOI:** 10.1016/j.mcpro.2025.101008

**Published:** 2025-06-02

**Authors:** Laura Cobos-Figueroa, Javier Robles-Parrado, Elisenda Alari-Pahissa, Begoña Galocha, Carmen Mir, Ana Pintor-Poveda, Eilon Barnea, Arie Admon, Pilar Lauzurica, Elena Lorente

**Affiliations:** 1 Centro Nacional de Microbiología, Instituto de Salud Carlos III, Madrid, Spain; 2 Hospital del Mar Medical Research Institute, Barcelona, Spain; 3 Faculty of Biology, Technion-Israel Institute of Technology, Haifa, Israel; 4 S.G. Servicios Aplicados, Formación e Investigación, Instituto de Salud Carlos III, Madrid, Spain

**Keywords:** HLA-B∗27, HLA-B∗14, ankylosing spondylitis, peptide repertoires, mass spectrometry, arthritogenic peptides, cross-reactivity

## Abstract

The human major histocompatibility complex class I gene *HLA-B∗27* is the main risk factor for ankylosing spondylitis (AS) through a mechanism that remains unknown. In African populations, where B∗27 is rare, the B∗14:03 allotype is strongly associated with AS, whereas B∗14:02, which differs at only one residue (L156R), is not associated. Using large-scale mass spectrometry–based peptide sequencing, we analyzed the peptidomes of HLA-B∗14:03, HLA-B∗14:02, and HLA-B∗27:05, obtaining more than 2000 ligands for each. Remarkably, we identified 1011 peptides shared by the AS-associated HLA-B∗27:05 and B∗14:03 alleles but not by the non-AS-associated B∗14:02 allele. Surprisingly, although B∗14:03 and B∗27:05 differ by 15 amino acids in their peptide-binding domain, they share a large portion of their ligands (64 and 43%, respectively), while B∗14:03 and B∗14:02, differing by only one residue, show less overlap (33–35%). The B∗14:03 peptide repertoire most closely resembles that of B∗27:05 at the P1, P2, and P5 peptide positions but diverges at the C-terminus, where B∗14:03 is more selective. Structural modeling suggests that the L156R difference between B∗14 alleles may induce long-range effects on peptide binding at P1, P2, and P5 residues, explaining the distinct repertoires. Most of the 1011 shared ligands contained R/K/A/G at P1, R at P2, and L/F at the C-terminus. Of these, ten peptides were previously identified as ligands of the three HLA-B∗27 subtypes most strongly associated with AS and are absent in nonassociated subtypes, while four peptides from the HLA 169 to 181 region—previously implicated in AS pathogenesis—were also identified, suggesting that differential peptide binding may influence disease development. In summary, the AS-associated allotypes B∗14:03 and B∗27:05, but not the non-AS-associated allotype B∗14:02, share similar peptide repertoires and binding characteristics, supporting specific common peptide ligands of HLA-B∗27:05 and B∗14:03 as a mechanism to explain the development of AS.

## Highlights

• AS-linked HLA-B∗14:03 and HLA-B∗27:05 share many ligands despite structural differences.
• Non-AS HLA-B∗14:02 shows distinct binding vs. AS-linked B∗14:03 and B∗27:05.
• 1011 ligands in B∗14:03/B∗27:05, absent in B∗14:02, suggest a common AS mechanism.
• One change at residue 156 in B∗14 may cause long-range effects on peptide binding.

## Graphical abstract

**Figure:**
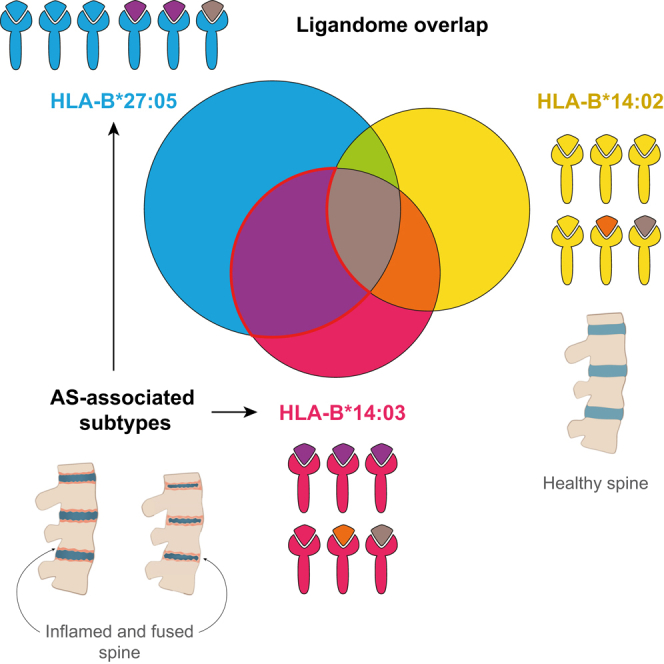

## In Brief

Ankylosing spondylitis (AS) is strongly associated with HLA-B∗27 but, in African populations, HLA-B∗14:03 also confers risk, unlike its close variant B∗14:02. Using mass spectrometry, we identified 1011 peptide ligands shared between AS-associated B∗14:03 and B∗27:05 but absent in B∗14:02. These ligands share key binding characteristics, suggesting a common peptide-driven mechanism for AS susceptibility. Our findings provide insight into the molecular basis of AS and the role of HLA–peptide interactions in disease development.

Spondyloarthropathies constitute a group of inflammatory diseases that typically affect the axial skeleton and include ankylosing spondylitis (AS) as a prototype for these disorders. The development of AS is believed to be influenced by both environmental and genetic factors. Among the environmental factors, the gut microbiome has gained increasing significance in explaining AS pathogenesis (1, 2, 3). A crucial genetic risk factor for AS is the human leukocyte antigen (HLA)-B∗27, which is expressed in approximately 90% of patients with AS, but only found in about 5% of the global population (4, 5).

Through population studies, a differential association of B∗27 subtypes with AS has been observed. The most commonly associated subtypes include HLA-B∗27:02 in Mediterranean populations, B∗27:04 in Chinese populations, and B∗27:05 in Caucasians and Native Americans, while B∗27:06 and B∗27:09 appear to have either weak or no association with the disease (6, 7, 8). Interestingly, in African populations, where the presence of the B∗27 allotype is rare, the B∗14:03 allotype has been strongly linked to AS (9, 10). However, the subtype B∗14:02, widely distributed globally, has not been associated with the disease. Notably, B∗14:02 differs from B∗14:03 only at residue 156 (L at B∗14:02 and R at B∗14:03). Additionally, other HLA-B alleles, such as B∗38, B∗39, B∗40, and B∗52, also seem to confer a higher susceptibility to AS (11).

The mechanism responsible for the differential association of HLA-B alleles with AS remains unclear. Several hypotheses have been proposed to explain the strong association of B∗27 with AS. On one hand, the B∗27 misfolding hypothesis proposed that the accumulation of aberrantly folded B∗27 heavy chains could induce endoplasmic reticulum (ER) stress and activate the unfolded protein response, resulting in the release of proinflammatory cytokines (12). However, the misfolded heavy chain of both B∗14:02 and B∗14:03 subtypes remained in the ER with a half-life similar to that of B∗27:05 (13) arguing against that hypothesis. On the other hand, the homodimer theory is based on the ability of the B∗27 molecule to form heavy chain homodimers through disulfide bonds of free cysteines such as ^67^C (14). These homodimers can be recognized on the cell surface by different receptors present on T and NK cells and could lead to their activation (15). However, although to a lesser extent than in B∗27:05, the formation of heavy chain homodimers has also been observed in both B∗14:03 and B∗14:02 (13), arguing against the homodimer hypothesis. Finally, the arthritogenic peptide hypothesis suggests molecular mimicry between pathogen-derived and self-peptides triggers a CD8^+^ T cell response leading to AS (16). One possibility of this hypothesis is that different AS-associated HLA-I alleles can present the same arthritogenic pathogen-derived and self-peptides. This would be based on highly similar qualitative peptide presenting capacity, which would be reflected by a structural similarity in peptide binding and highly overlapping peptide repertoire among arthritogenic alleles that would be different from those of non-arthritogenic subtypes. Perhaps in agreement with this hypothesis, the crystal structures of B∗14:02, B∗27:05, and B∗27:09 showed that B∗14:02 displayed a distinct conformation when presenting an Epstein-Barr virus–derived peptide compared to the 2 B∗27 subtypes. However, the presentation of an endogenous cathepsin A–derived peptide was very similar across all three HLA subtypes despite differences in the binding grooves between B∗14:02 and B∗27:05/B∗27:09 (17).

Regarding the study of peptide repertoire of AS-associated alleles, a previous work analyzed by mass spectrometry the *m/z* spectra of the peptides eluted from AS-associated and non-associated B∗14 subtypes expressed on a lymphoblastoid cell line and compared it to that of B∗27:05 (18). Among the hundreds of peaks detected for the different alleles, it was estimated that only a small proportion consisted of peptides common between B∗27:05 and HLA-B∗14:03 and that these peptides were also common with the non-arthritogenic HLA-B∗14:02. About 20 selected peaks per allele of shared and non-shared peptides were further sequenced by tandem mass spectrometry, which did not identify any peptide ligand specific of AS-associated alleles. Nevertheless, this study indicated clear differences in the peptide binding profiles of the B∗14:03 and B∗14:02.

In the present study, we aimed at assessing and comparing the peptide repertoires of B∗14:02, B∗14:03, and B∗27:05 expressed in the same lymphoblastoid cell line by unbiased large-scale sequencing by tandem mass spectrometry. The high number of peptides sequenced allowed us to improve the structural binding characteristics of the different alleles, to observe the high degree of overlap between the arthritogenic alleles B∗14:03 and B∗27:05 and, importantly, to find 1011 peptide ligands shared exclusively by the two arthritogenic alleles B∗27:05 and B∗14:03 and absent from ligandome of the non-arthritogenic B∗14:02 allele.

## Experimental Procedures

### Cell Lines

The human lymphoblastoid cell line HMy2.C1R (C1R) expresses low levels of HLA molecules (HLA-C∗04:01 and HLA-B∗35:03) on the cell surface (19). C1R transfectants expressing B∗14:02, B∗14:03, or B∗27:05 have been previously described (18, 20). Cell lines were cultured in RPMI 1640 medium supplemented with 2 mM L-glutamine, 7% fetal bovine serum (Gibco, Thermo Fisher Scientific), penicillin, and streptomycin.

### Isolation of HLA-B∗14:02-, B∗14:03-, and B∗27:05-bound Peptides

HLA-B∗14- and B∗27-bound peptides were isolated from three independent preparations of 1 × 10^9^ cells as previously described (21). In addition, three independent preparations of 1 × 10^9^ untransfected C1R cells were obtained to account for the ligands of the endogenous HLA of this cell line. Cells were lysed using 150 mM NaCl, 20 mM Tris–HCl, pH 7.5, 1% Igepal CA-630 (Sigma-Aldrich) buffer containing protease inhibitor cocktail (Roche). After centrifugation, the supernatant was passed first through a precolumn with CNBr-activated Sepharose 4B beads (GE Healthcare) to remove nonspecific interactions and then through a column, containing W6/32 (a pan-HLA-I–specific antibody) bound to CNBr-activated Sepharose beads. Next, the columns were successively washed with 20 mM Tris–HCl, pH 8.0, containing: 1) 150 mM NaCl; 2) 400 mM NaCl; 3) 150 mM NaCl; and 4) buffer without NaCl. The HLA-bound peptides were eluted with 1% TFA (Sigma-Aldrich), filtered through a Vivaspin 2 filter (cut-off 5000 Da) (Sartorius Stedim Biotech), and concentrated in a SpeedVac (Savant; DJB Labcare).

### Mass Spectrometry Analysis

Samples were analyzed by LC-MS/MS with a Q-Exactive-Plus mass spectrometer (Thermo Fisher Scientific) as previously described (22). The peptides were resolved with a 7 to 40% acetonitrile gradient with 0.1% formic acid for 180 min and 0.15 μl/min on a capillary column pressure-packed with Reprosil C18-Aqua (Dr Maisch, GmbH) as previously described (23). The dynamic exclusion was set to 20 s. The selected masses were fragmented from the survey scan of the *m/z* 300 to 1800 AMU at a resolution of 70,000. Tandem mass spectrometry (MS/MS) spectra were acquired beginning at *m/z* 200 with a resolution of 17,500. The target value was set to 1 × 10^5^ and the isolation window to 1.8 *m/z*. The study was done at TopN = 10 and a collision energy of 25. Peptide sequences assignation from the MS/MS spectra were done as described below.

The mass spectrometry data have been deposited to the MassIVE repository (http://massive.ucsd.edu) with the dataset identifier MSV000095860 (ftp://MSV000095860@massive.ucsd.edu, password: EL_2024).

### Analysis of HLA-B∗14:02-, B∗14:03-, and B∗27:05-bound Peptides

The peptide pools from each of the three independent preparations of HLA-B∗14:02, -B∗14:03, and -B∗27:05 transfectants, along with the parental cell line, were separately subjected to MS analysis. Peptides were identified from the MS/MS spectra using the MaxQuant software (version 1.5.8.3, https://www.maxquant.org) (24) with the Andromeda search engine (25) and the human UniProt/SwissProt database (73,101 entries) under the following parameters: precursor ion mass and fragment mass tolerance 20 ppm, false discovery rate 0.01, and peptide-spectrum matching false discovery rate 0.05. We included oxidation (Met), acetylation (protein N terminus), and Gln to Pyro-Glu conversion as variable modifications, while no fixed modifications were considered. Additionally, no protease was selected in the MaxQuant search. Identifications derived from the reverse database and known contaminants were excluded.

The sequenced peptides eluted from the transfectants were selected based on the following criteria: 1) peptides ranging from 8 to 12 residues in length, which is the length range of the majority of MHC-I ligands, 2) peptides absent in untransfected C1R cell line preparations, 3) peptides not previously identified in another study with untransfected C1R cells (26). Peptides fulfilling these criteria included the vast majority of the sequenced peptides and were used for the subsequent analyses. No filter based on anchor motifs or theoretical affinity was included.

For quantitative analyses, the intensity of every ion peak was normalized to the sum of the intensities of all the selected ligands of that elution. Then, for each transfectant, the normalized intensities of the equivalent ion peaks from the three individual experiments were averaged.

### Experimental Design and Statistical Rationale

Peptides bound to HLA-B∗14:02, HLA-B∗14:03, and HLA-B∗27:05 were isolated from three independent technical replicates of 1 × 10^9^ transfected cells per condition, following established protocols. Additionally, peptides from three independent preparations of 1 × 10^9^ untransfected C1R cells were analyzed as controls to account for ligands derived from endogenous HLA molecules. Technical triplicates help assess the reproducibility of the analysis and minimize experimental variability.

Differences in length and in residue frequencies were analyzed using the χ^2^ test with Bonferroni correction when applicable. Relative molecular mass or intensity differences among peptide sets were assessed by an unpaired *t* test.

## Results

### Highly Overlapping Peptide Repertoire Among AS-Associated HLA Alleles

**Fig. 1:**
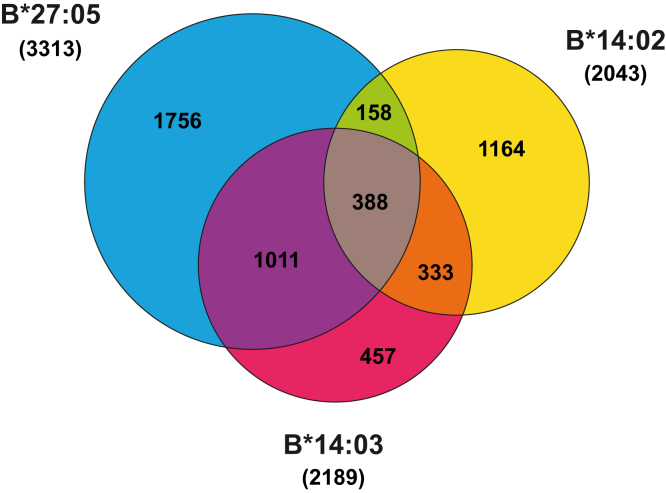
**Venn diagram of the peptides identified in HLA-B∗27 and in the two HLA-B∗14 subtypes**. The identified peptides bound to HLA-B∗27:05 (*blue*), HLA-B∗14:02 (*yellow*), and HLA-B∗14:03 (*garnet*) are depicted. Overlapping sections show the peptides identified in more than one HLA allele. HLA, human leukocyte antigen.

To compare the repertoires of peptides bound naturally to the AS-associated alleles HLA-B∗27:05 and -B∗14:03 among them and also with that of the non-AS–associated B∗14:02 allele, we eluted and analyzed the peptides bound to HLA-B∗14:02, -B∗14:03, and -B∗27:05 molecules expressed in the lymphoblastoid cell line C1R through lysis of the transfectants, HLA-I affinity purification with the anti-pan-HLA-I Ab W6/32, acid extraction of the bound peptides, high-throughput sequencing by tandem mass spectrometry, and MaxQuant software processing of three independent preparation from each HLA-B subtype. Peptides ranging from 8 to 12 amino acids in length, found in any of the three preparations but not present in our dataset or in previously published eluates from untransfected C1R cells, were considered as the peptidome of the given HLA-B subtype. In this way, we identified 2043, 2189, and 3313 peptide sequences linked to HLA-B∗14:02, -B∗14:03, and -B∗27:05 molecules, respectively (Fig. 1 and Supplemental Table S1). Between 12 and 19% of the identified peptides sequences were presented by the three allotypes. The overlap between the peptide repertoires of the B∗14 subtypes was only ∼33 to 35%. Approximately 27% of the B∗14:02 ligands and 16% of the B∗27:05 ligands were common between both allotypes. Interestingly, 1399 common peptides among the repertoires of B∗14:03 and B∗27:05 were identified, 1011 of them being absent from the B∗14:02 peptidome, representing 64% and 42% of the B∗14:03 and B∗27:05 ligands, respectively. These results indicate that B∗14:02 exhibits a substantially distinct peptide repertoire compared to B∗14:03 and B∗27:05, despite its structural similarity with B∗14:03. It is noteworthy that the peptide repertoires of the two subtypes associated with AS do display a significant degree of overlap due to shared ligands that are absent from the peptidome of the non-arthritogenic subtype B∗14:02.

### Different Size of the Peptides Bound to HLA-B∗27:05, -B∗14:02, and -B∗14:03

**Fig. 2:**
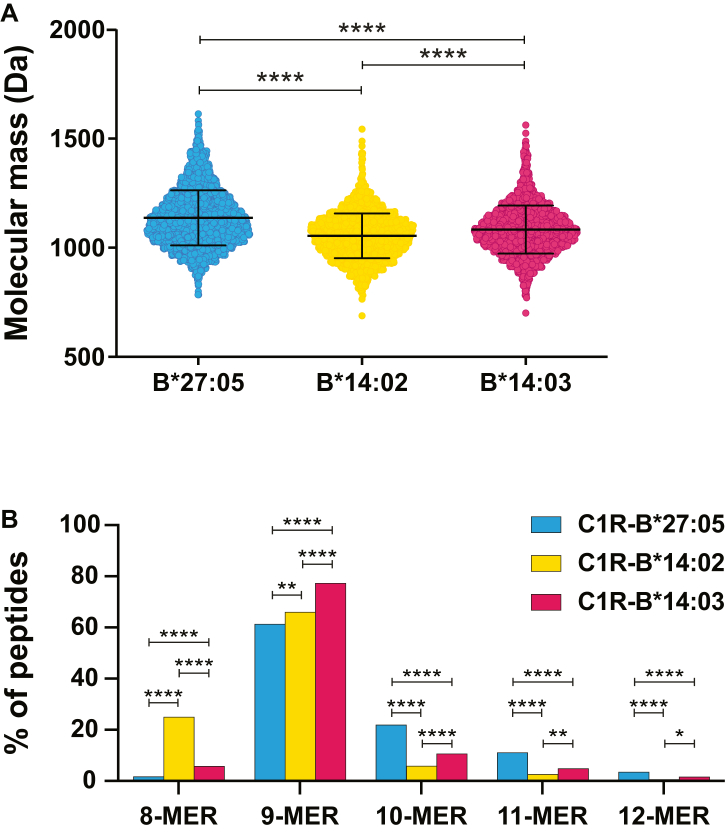
**Molecular weight and length of the peptides presented by HLA-B∗27 and B∗14 subtypes**. MW (*A*), and length (*B*) of the peptides eluted from the different HLA molecules, B∗27:05 (*blue*), B∗14:02 (*yellow*), and B∗14:03 (*garnet*). Statistically significant differences were estimated with the *t* test (*A*) and χ^2^ test (*B*). They are labeled with *asterisks* (∗) and their *p* values are ns for *p* > 0.05; ∗ for *p* ≤ 0.05; ∗∗ for *p* ≤ 0.01; ∗∗∗ for *p* ≤ 0.001, and ∗∗∗∗ for *p* ≤ 0.0001. HLA, human leukocyte antigen.

The average molecular mass of the peptides bound to B∗27:05 was significantly higher (1138 Da) than that of the B∗14:02 or B∗14:03 ligands (1056 and 1087 Da, respectively) (Fig. 2*A*). The length distribution of the bound peptides differed between allotypes. Although all of them bound mostly nonamers, B∗27:05 showed a higher affinity for ligands of 10 or more residues (more than 30% of the ligands) and B∗14:02 for octamers (more than 20% of the ligands) than the other allotypes (Fig. 2*B*). The percentage of nonamers in the HLA-B∗14:03 peptidome was significantly higher than in the peptidomes of the other allotypes. These results indicate that B∗27:05 binds peptides of larger size and molecular weight than B∗14 subtypes, that B∗14:02 has a higher affinity for octamers than the other two allotypes, and that B∗14:03 has an intermediate behavior with a higher affinity for nonamers.

### Differences in Sidechain Composition Between the B∗14 and B∗27:05-Bound Peptide Repertoires

**Fig. 3:**
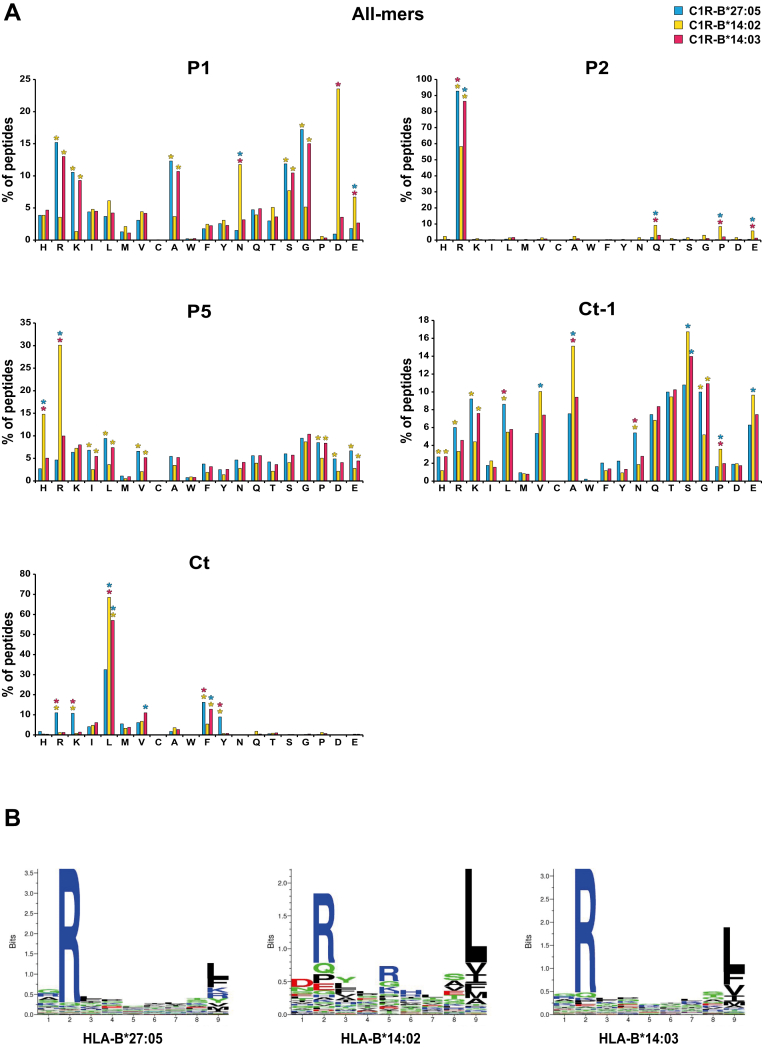
**Amino acid frequencies of peptides eluted from the different allotypes**. *A*, amino acid frequencies at P1, P2, P5, Ct-1, and at C terminus. Percentage of peptides with each amino acid residue in each position for the HLA-B∗27:05, B∗14:02, and B∗14:03 peptidomes (*blue*, *yellow*, and *garnet bars*, respectively). Statistically significant differences were estimated with the χ^2^ test with Bonferroni correction. ∗*p* < 0.01. The color of the asterisk implies significance with respect to the peptidome represented by that color. *B*, sequence motifs generated by GibbsCluster (https://services.healthtech.dtu.dk/services/GibbsCluster-2.0/). HLA, human leukocyte antigen; Ct, C terminal.

**Supplemental Figure S1:**
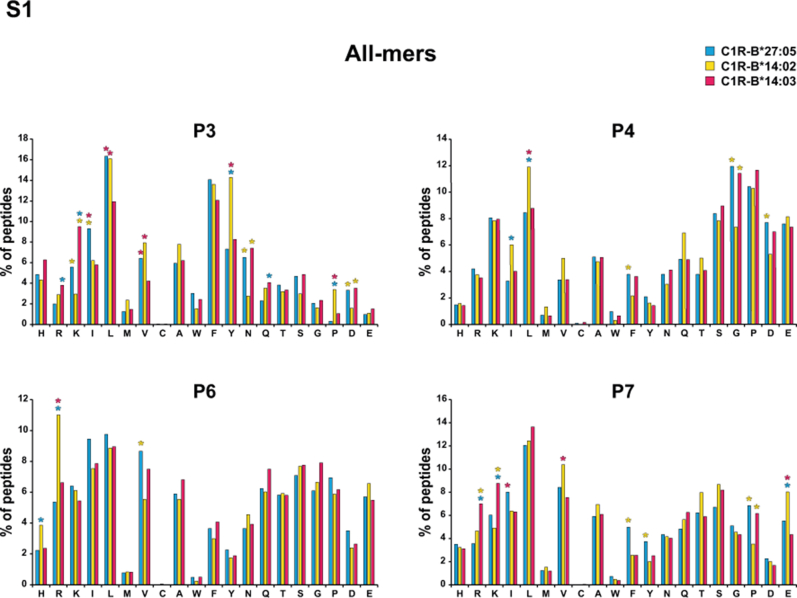


**Supplemental Figure S2:**
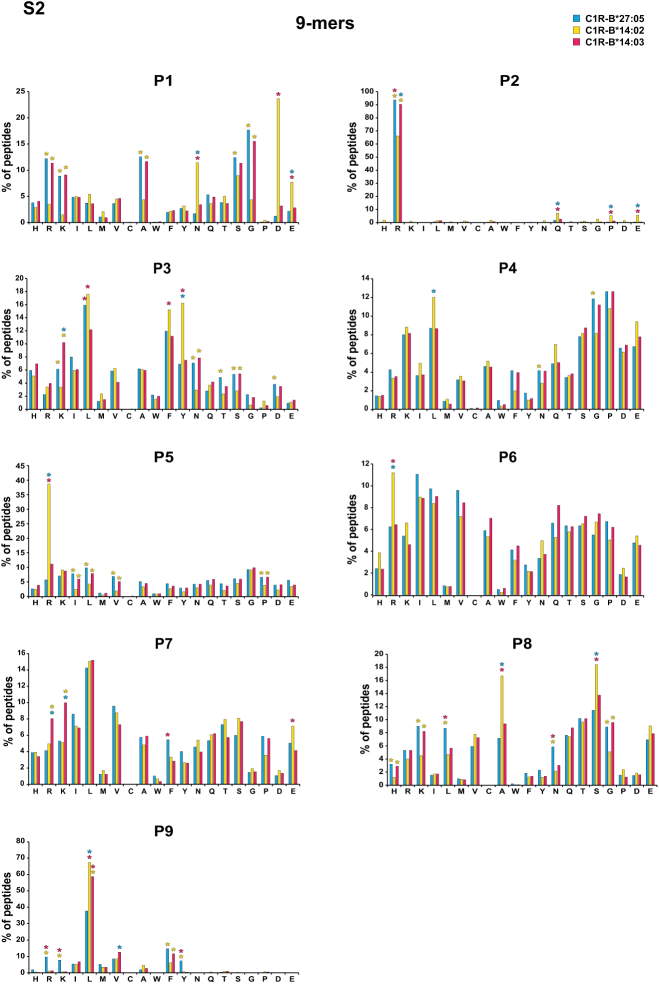


To characterize the preferential amino acid motifs of each allotype, we analyzed the frequency of each amino acid residue at each position both among the complete repertoire and in nonamers, since they provided the highest number of peptides of the same size, which allowed a more powerful statistical analysis (Fig. 3, Supplemental Figs. S1 and S2). There were significant differences between the allotypes in all the analyzed positions, although the greatest changes occurred in P1, P2, P5, C terminal (Ct)-1, and Ct (Fig. 3 and Supplemental Fig. S2) rather than in the rest of positions (Supplemental Figs. S1 and S2). The percentage of the peptides with the same residues in P1 was very similar between B∗27:05 and B∗14:03, with R, K, A, G, and S (basic and small) as predominant residues, while B∗14:02 differed greatly from the previous ones, presenting an increase in acidic residues D and N (Fig. 3 and Supplemental Fig. S2). More than 80% of the peptides bound to the AS-associated subtypes contained R at the P2 anchor position, whereas this residue was only present in approximately 60% of peptides bound to B∗14:02. Instead, significant number of the B∗14:02 ligands had Q, P, and E at P2. At P5, no significant changes were observed in the percentages of usage of the different residues between B∗27:05 and B∗14:03, but a significant percentage of the B∗14:02-bound peptides had R at this position, which could be another anchor motif for this allotype. In this sense, it was noted that while only 34% of the B∗14:02 nonamers with R at P5 also presented R at P2, almost 90% of those without R at P5 presented it at P2. At the last-but-one Ct position (Ct-1; P8 in nonamers), different preferential residues were observed between allotypes associated with AS and B∗14:02. However, the Ct (P9 position in nonamers) was where the two allotypes associated with AS differed the most: while B∗27:05 presented peptides with varying types of residues at this position (basic and hydrophobic), B∗14:03 mostly bound peptides with aliphatic residues, mainly L. In this position, the ligands of B∗14:02 had a greater similarity with those of B∗14:03 than in other positions. Together, all these data show a high similarity in the amino acid composition of B∗27:05 and B∗14:03 ligands in most positions except for Ct, where B∗14:03 seemed to be more restrictive than B∗27:05 and bound mostly aliphatic amino acids. The greatest differences in the peptides presented by the non-AS–associated subtype B∗14:02, compared to the two previous allotypes, were found at positions P1, P2, P5, and Ct-1.

### The Single Amino Acid Difference Between the Sequences of HLA-B∗14:02 and HLA-B∗14:03 Could Affect Amino Acid Frequencies at Positions P1, P2, and P5 of Their Peptide Ligands

**Fig. 4:**
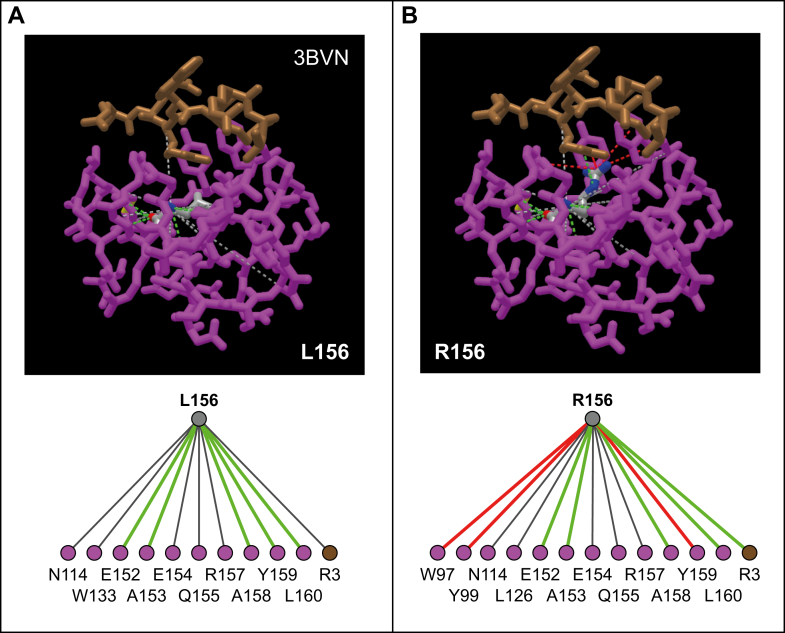
**Interaction network between residue 156 and others positions by mutation analysis with iCn3D** (https://www.ncbi.nlm.nih.gov/Structure/icn3d/full.html) **on the 3D structure of HLA-B∗14:02 from 3BVN**. Interactions of residue L156 (*A*) or predicted chemical interactions of R156 (*B*) of the HLA structure with other positions of the same molecule and with the viral peptide (RRRWRRLTV) with which it was crystallized. In the structures, residue 156 is represented in *gray*, the rest of the HLA residues with which it interacts in *magenta*, and the peptide in *brown*. Below, summary of the direct interactions between residue L156 (*A*) or R156 (*B*) and the other HLA residues (*magenta balls*) or the peptide (*brown ball*) residues. *Green*, *red*, and *gray lines* represent hydrogen bonds, π-cation, and contacts, respectively. HLA, human leukocyte antigen.

**Fig. 5:**
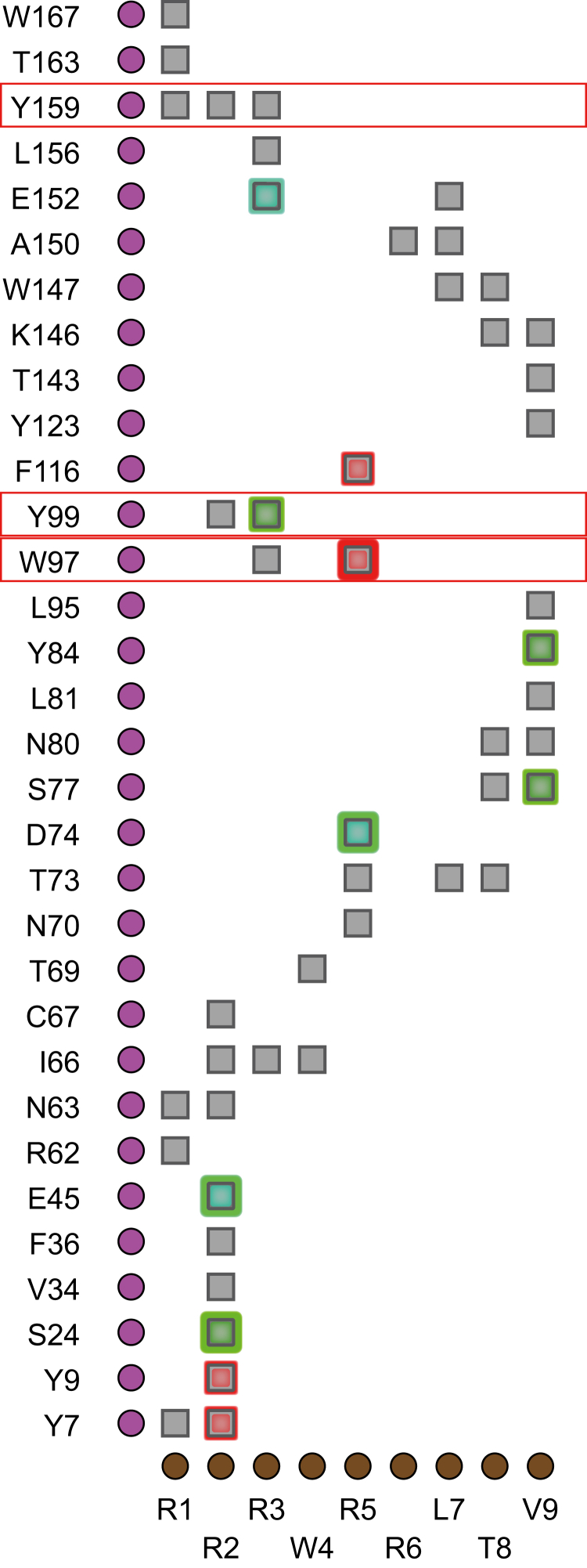
**Scatterplot of interactions between 3BVN_A (HLA) and 3BVN_C (peptide) chains**. Interactions between different HLA residues and the viral peptide (RRRWRRLTV) from HLA-B∗14:02 crystallography (3BVN). *Green*, *red*, *cyan*, and *gray squares* represent hydrogen bonds, π-cation, salt bridges, and contacts, respectively. Figure obtained with iCn3D. HLA, human leukocyte antigen.

The fact that the peptide signature of B∗14:03 differed the most from that of B∗14:02 at P1, P2, and P5 positions was unexpected, since these 2 B∗14 molecules differ only in residue 156 (B∗14:02^156^L, B∗14:03 ^156^R), which is part of the D and E binding pockets of HLA and is expected to have direct influence only on the central region of the bound peptide, specifically on P3. Thus, we studied whether residue 156 could have a long-range indirect effect on residues that could modulate P1 and P2 and P5 residue frequency. For this we utilized the crystallographic structure of B∗14:02 in complex with the viral peptide pLMP2 peptide (3BVN) (17) and employed the iCn3D program (Web-based 3D Structure Viewer-NCBI) (27) to virtually mutate the L^156^ residue to R^156^. This mutation significantly impacted direct and indirect interactions with various positions of the peptide (Figs. 4 and 5). The main changes were observed in the interaction of residue 156 with P3, as expected, but also in the interaction of residues W97, Y99, and Y159 with positions P1, P2, P3, and P5 (Fig. 5). Of note, a cationic interaction between the W97 residue and P5 of the peptide present in B∗14:02 may be hindered or reduced by a new intramolecular interaction of W97 with R156 in B∗14:03, which could explain the absence of the putative secondary anchor motif of R at the P5 position of its peptide ligands. Altogether, these results offer a plausible explanation for the dissimilar amino acid preference distribution observed between the 2 B∗14 subtypes at P1, P2, P3, and P5 positions.

### Differential Characteristics Between Common and Specific Peptide Ligands

According to the arthritogenic peptide hypothesis, the arthritogenic peptides should be found only within the subgroup of peptides common to the two allotypes associated with AS and absent from the non-AS–associated allotype. Thus, we assessed the specific characteristics of this subgroup and compared them with those of the subgroups presented by one, two, or the three alleles.

**Fig. 6:**
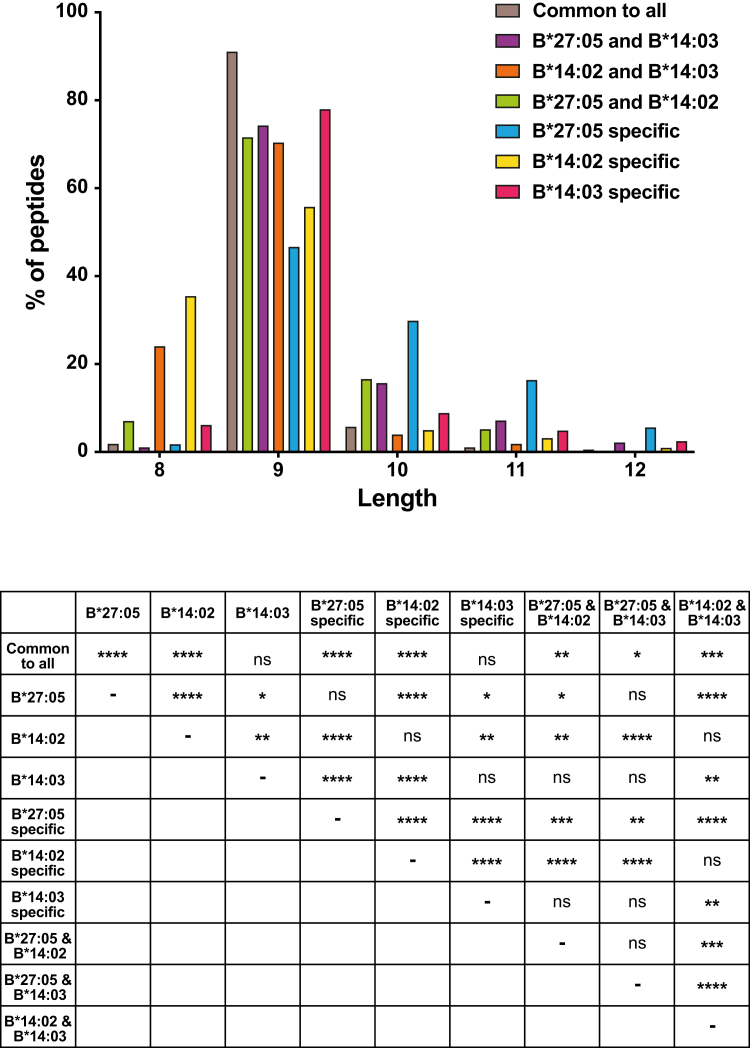
**Length distribution of the peptides differentially bound to the three alleles.** Percentage of peptides with the indicated length from the total of peptides belonging to each of the subgroups analyzed: common peptides between the three HLA alleles (*brown*), between B∗27:05 and B∗14:02 (*green*), between B∗27:05 and B∗14:03 (*purple*), between B∗14:02 and B∗14:03 (*orange*), and those specific of B∗27:05 (*blue*), B∗14:02 (*yellow*), and B∗14:03 (*garnet*) peptidomes. The table shows the statistical significance between the size distribution of the different subgroups and the global peptides identified for each HLA molecule, which were estimated with the χ^2^ test. *p* values are represented as ns for *p* > 0.05; ∗ for *p* ≤ 0.05; ∗∗ for *p* ≤ 0.01; ∗∗∗ for *p* ≤ 0.001, and ∗∗∗∗ for *p* ≤ 0.0001. HLA, human leukocyte antigen.

As expected, the length distribution differed between the subgroups (Fig. 6). The limited capacities of B∗27:05 to bind octamers and of B∗14:02 to bind peptides longer than nine amino acids (Fig. 2) enriched the set of overlapping peptides in nonamers and diminished the percentages of specific nonamers of these alleles. The ligands common to the three allotypes significantly presented the highest percentage of nonamers (91%). No significant differences in length were observed between the overlapping peptides of B∗27:05/B∗14:03 and those of B∗27:05/B∗14:02, those specific for B∗14:03, or with the total of B∗14:03 or B∗27:05-bound peptides.

**Fig. 7:**
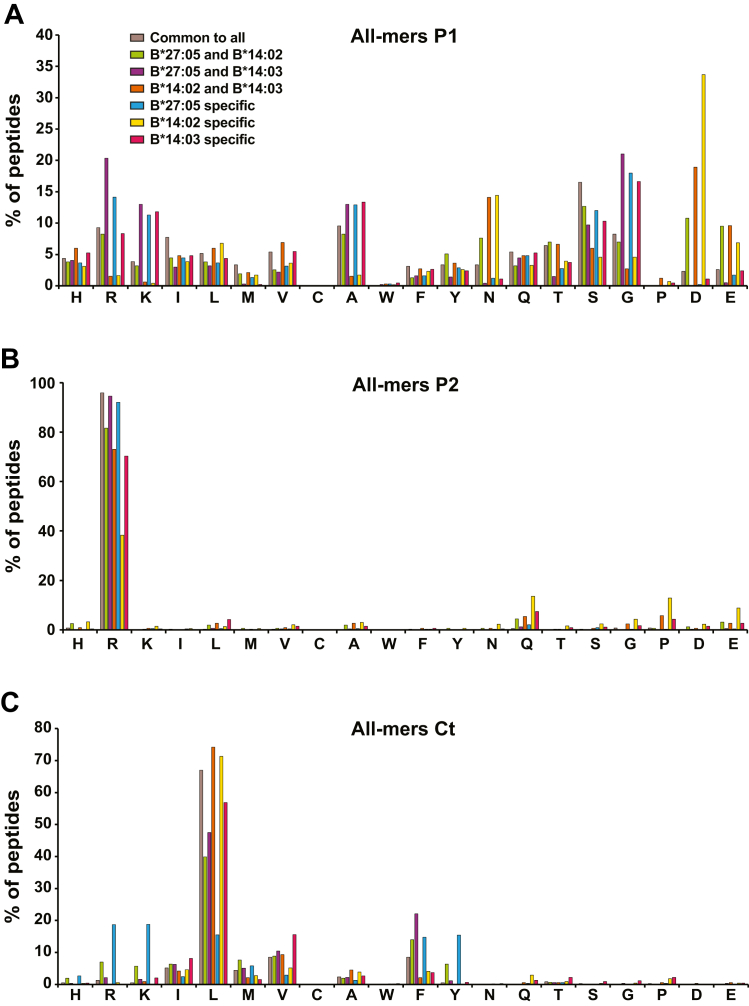
**Amino acid frequencies at P1, P2, and Ct of the peptides differentially bound to the three alleles.** Percentage of peptides with the indicated residue in P1 (*A*), P2 (*B*), and Ct (*C*) positions of peptides belonging to each of the subgroups analyzed: common peptides between the three HLA alleles (*brown*), between B∗27:05 and B∗14:02 (*green*), between B∗27 and B∗14:03 (*purple*), between B∗14:02 and B∗14:03 (*orange*), and those specific of B∗27:05 (*blue*), B∗14:02 (*yellow*), and B∗14:03 (*garnet*). The statistical analysis for these figures can be found in Supplemental Figure S13. HLA, human leukocyte antigen; Ct, C terminal.

**Supplemental Figure S3:**
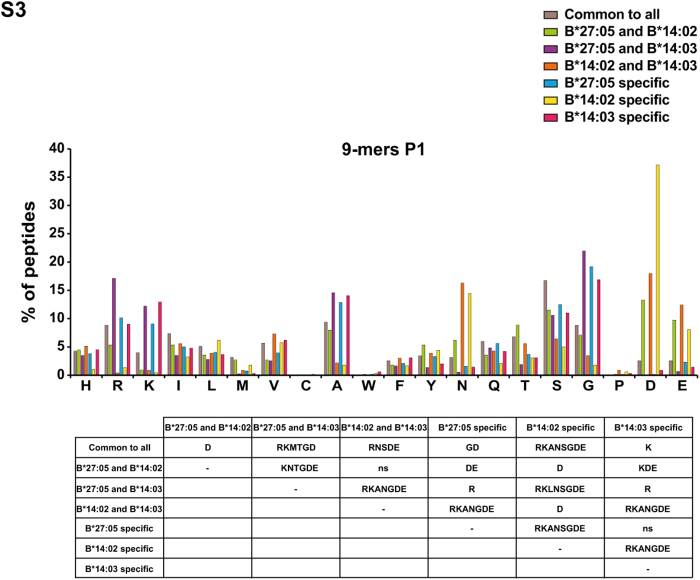


**Supplemental Figure S4:**
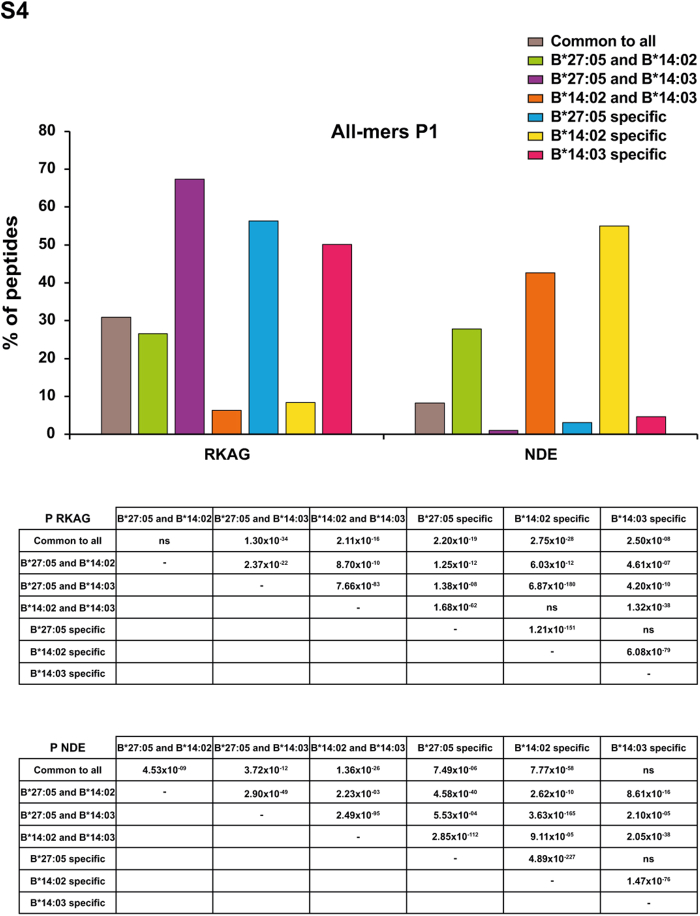


**Supplemental Figure S5:**
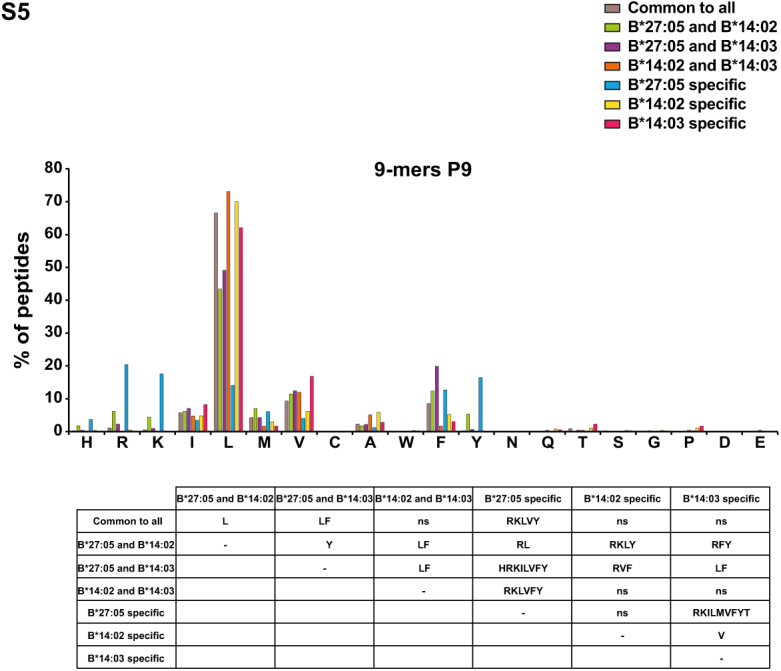


**Supplemental Figure S6:**
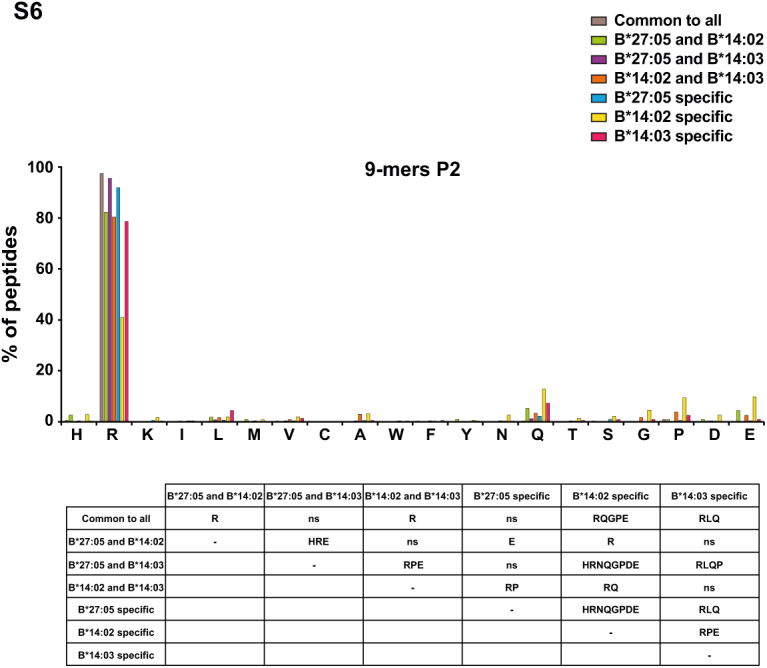


**Supplemental Figure S7:**
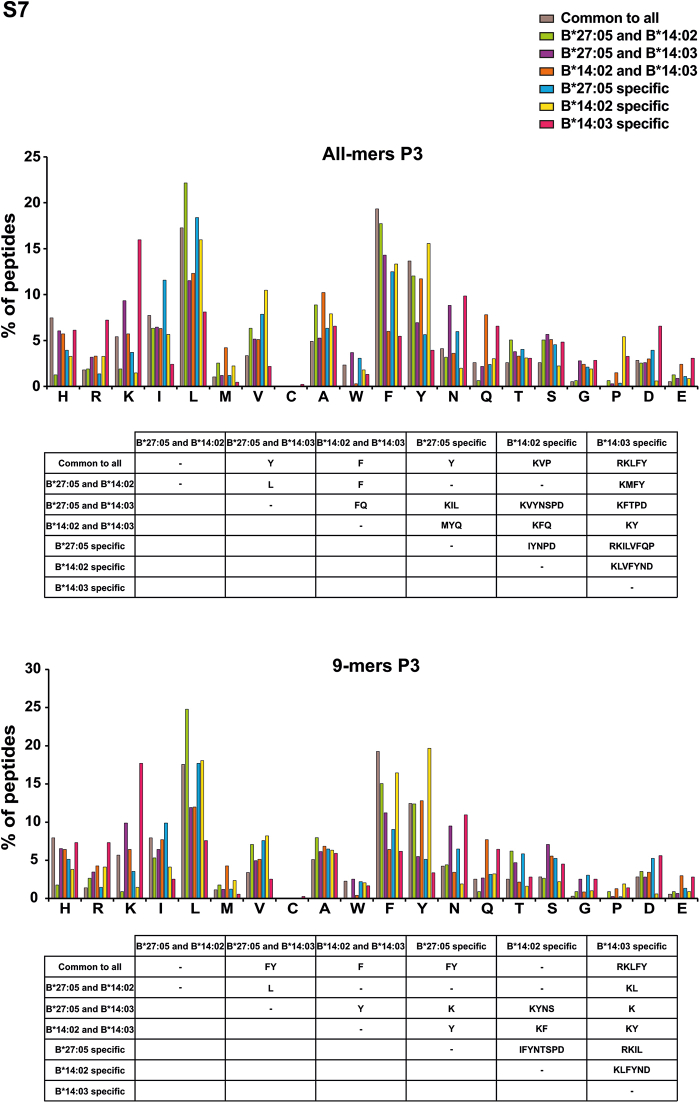


**Supplemental Figure S8:**
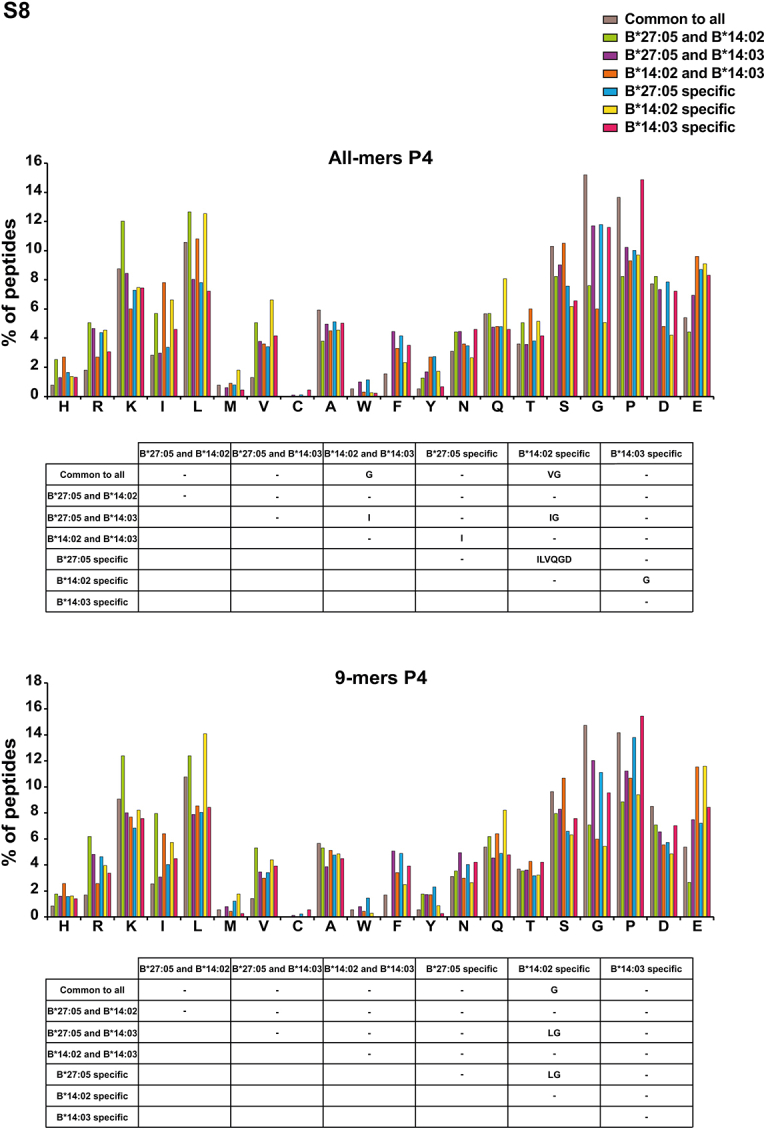


**Supplemental Figure S9:**
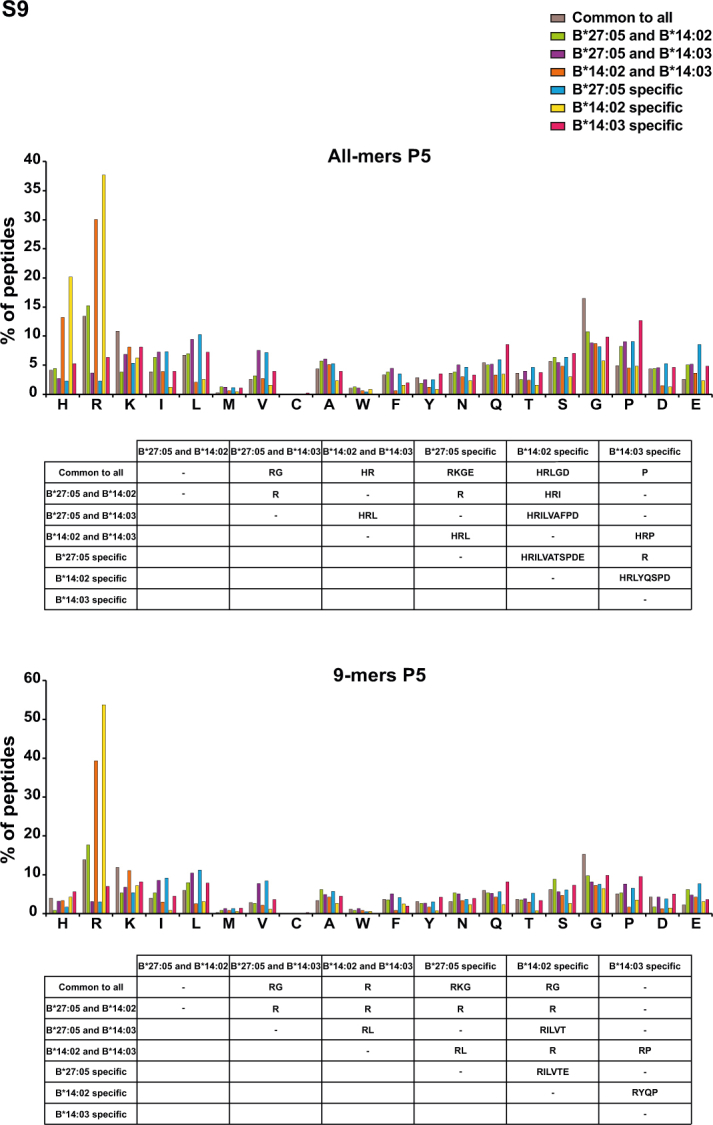


**Supplemental Figure S10:**
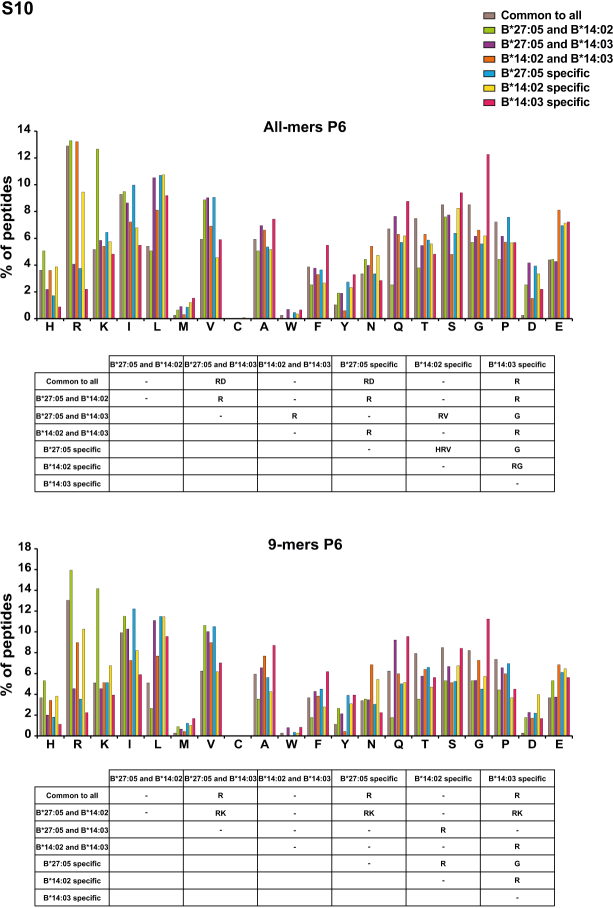


**Supplemental Figure S11:**
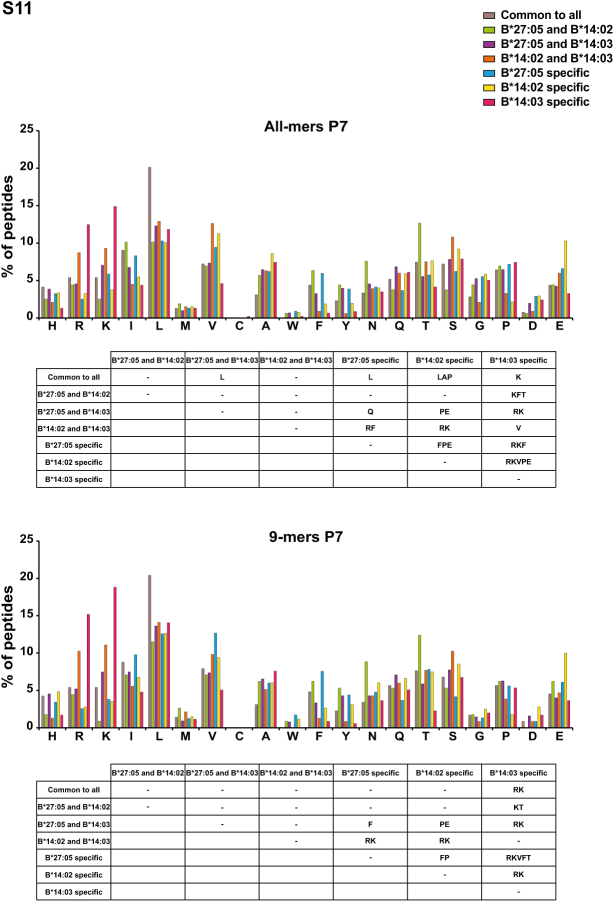


**Supplemental Figure S12:**
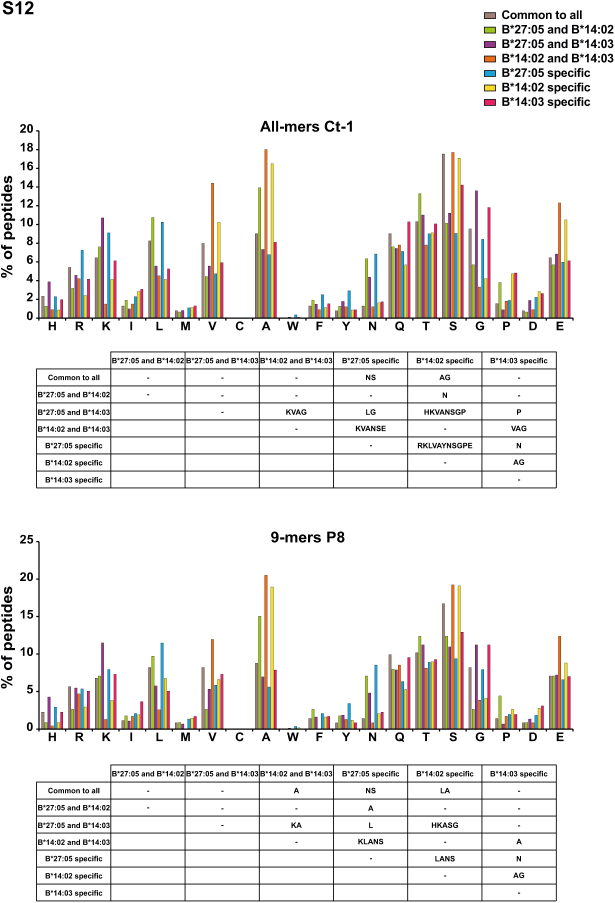


The amino acid frequencies at each position of the different subgroups were compared. At P1, (Fig. 7*A* and S3) the enrichment in basic residues (R and K) and small nonpolar amino acids (A and G) observed previously in the B∗27:05 and B∗14:03 peptidomes was even more marked in the overlapping ligands between the two allotypes (67%) (Supplemental Fig. S4) and in the ones specific for B∗27:05 (56%) and B∗14:03 (50%). Conversely, the common ligands between B∗14:02 and B∗14:03 and those specific for B∗14:02 had a greater preference for peptides with acidic residues (D and E) and N at that position. The proportion of N, D, and E residues at P1 was very low (>5%) in the overlapping and specific ligands of HLA-B∗27:05 and B∗14:03, but increased up to 28%, 43%, and 55% in the overlapping peptides between B∗27:05 and B∗14:02, B∗14:02, and B∗14:03, and the peptides specific for B∗14:02, respectively. The ligands common to the three allotypes showed a greater diversity of residues at P1 than the rest of the subgroups. At P2, more than 90% of the peptides common to the three allotypes, to B∗27:05 and B∗14:03 and of those specific to B∗27:05 presented an R (Fig. 7*B* and Supplemental Fig. S6). This percentage was significantly reduced to 80% in the overlaps between B∗27:05 and B∗14:02, between B∗14:02 and B∗14:03, and the peptides specific to B∗14:03. Only 40% of the specific ligands of B∗14:02 presented R at P2, possibly because this molecule seems to have a second anchor motif in P5 that could replace that of P2. Ct/P9 was another position in which the ligand subgroups differed the most (Fig. 7*C* and Supplemental Fig. S5), due to the greater diversity of residues that B∗27:05 could bind to at this position. While more than 60% of the ligands bound by all three allotypes, by B∗14:02 and B∗14:03, and by only B∗14:02 or B∗14:03 presented L at P9, this percentage decreased to around 40 to 50% in the ligands shared by B∗27:05 and any of the B∗14 subtypes. Only around 15% of B∗27:05-specific peptides presented L at P9, with a significant increase in basic (R and K) and aromatic (F and Y) residues at this position. Other intermediate positions also showed differences between the diverse subgroups (Supplemental Figs. S7–S12), mainly at P3, at P5, where B∗14:02 has preference for R, and at Ct-1/P8, where the ligands that overlapped with those of B∗14:02 had an increase in frequency of A residues.

These results indicate that the main differential characteristics of the peptides common to the two allotypes associated with AS were found at P1, with the highest preference for RKAG residues, while those common to all three allotypes, to B∗27 and B∗14:02, to the 2 B∗14 subtypes, and those specific for B∗14:02 had relatively higher affinity for peptides with NDE at that position. In addition, differences at other peptide positions, mainly at P2 and Ct, were found not specific of the B∗27:05/B∗14:03.

### Common Peptides are More Abundant in the Eluates Than Specific Peptides

**Fig. 8:**
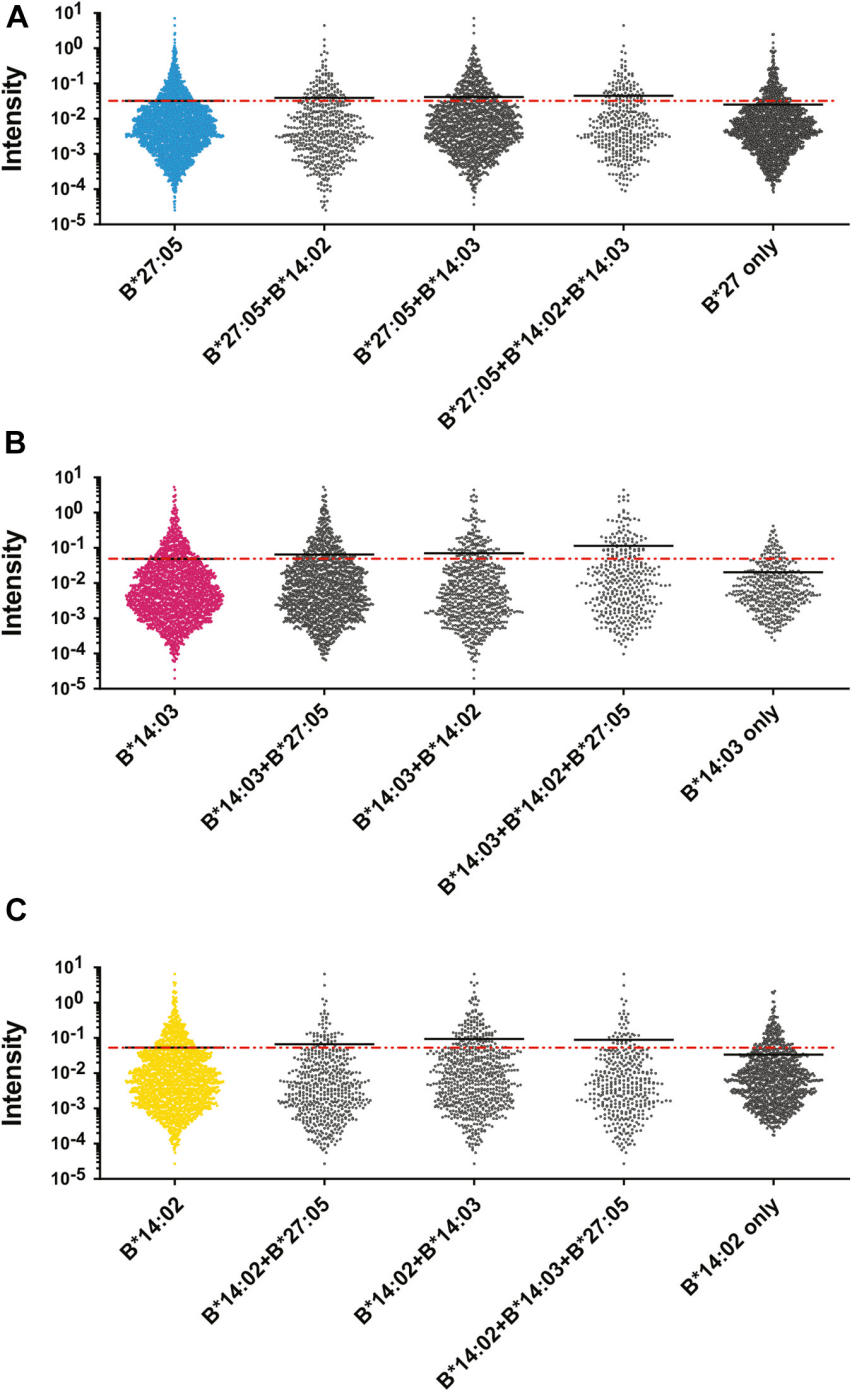
**Quantitative differences between the normalized intensities of the peptides differentially bound to the three alleles**. The intensity of any given ion peak was normalized to the total intensity of all the identified ligands for that HLA molecule. The mean normalized intensity of each ion peak from the three individual experiments for each cell line was taken as the amount of that peptide relative to the total amount of ligands identified in that cell line. *A*, intensity differences between the total peptides identified in HLA-B∗27:05 and the different subgroups. *B*, intensity differences between the total peptides identified in HLA-B∗14:03 and the different subgroups. *C*, intensity differences between the total peptides identified in HLA-B∗14:02 and the different subgroups. HLA, human leukocyte antigen.

It has been proposed that the induction of AS may also be influenced by the abundance of the presented arthritogenic peptides, which will depend in part on the peptide-binding characteristics of HLA-I molecules and on the availability of the peptides in the ER (28). In any case, this abundance can be reflected by the intensity of the peptide peaks in the mass spectrum. For each experiment, peak intensities were measured, normalized to the overall intensity of all peaks within that experiment, and then the normalized intensities of equivalent peaks among the three experiments for each HLA-B molecule were averaged (Fig. 8). Additionally, the percentage contributed by different subgroups to the overall intensity was determined (Table 1). The average intensity of the specific ligands of B∗27:05 was significantly lower than that of the common ones between this molecule and B∗14:03 and of those common to the three allotypes, 38/53, 41/31, and 17/12, respectively. Also, the specific ligands of B∗14:02 and B∗14:03 had a lower signal intensity than the rest of subgroups, while those presented by the three allotypes had a higher intensity (Table 1 and Fig. 8). The common peptides between B∗14:03 and any of the other two HLA molecules analyzed also showed, in general, a higher signal intensity than the B∗14:03-specific ligands, but less than that of the ligands common to the three allotypes.

**Table 1:** Comparison between the abundance and the percentage of ligands of each subgroup

	Number of ligands	HLA-B∗27:05		HLA-B∗14:02		HLA-B∗14:03	
		% Intensity	% Ligands	% Intensity	% Ligands	% intensity	% ligands
Common only B∗27:05 and B∗14:02	158	4	5	2	8		
Common only B∗27:05 and B∗14:03	1011	41	31			46	46
Common only B∗14:02 and B∗14:03	333			34	16	7	15
Common to all	388	17	12	34	19	44	18
Specific B∗27:05	1756	38	53				
Specific B∗14:02	1164			30	57		
Specific B∗14:03	457					3	21

The table shows the percentage of the total intensity and the percentage of the total ligands that each subgroup represents in the HLA-B∗27:05, -B∗14:02, and -B∗14:03 repertoires. The total number of peptides that are part of each subgroup is also indicated.

HLA, human leukocyte antigen.

Altogether, these data indicate that specific peptides of a single allotype are, on average, less abundant than common ligands.

### Differential Presentation of HLA-Derived Peptides

Our analysis identified several peptides derived from the HLA molecule itself, particularly from the 169 to 181 region of the mature protein, which were differentially presented by disease-associated and non-disease-associated HLA subtypes (Table 2). Interestingly, the peptides RRYLENGK, RRYLENGKE, RRYLENGKET, and RRYLENGKETL were detected exclusively in disease-associated subtypes (HLA-B∗27:05 and HLA-B∗14:03) and absent in the non-disease-associated HLA-B∗14:02. Furthermore, since the RRYLENGKETL sequence is not conserved in either B∗14:02 or B∗14:03 (RRHLENGKETL), its presence in the B∗14:03 peptide repertoire likely originates from other HLA molecules present in the C1R cell line that do contain it, such as B∗35:03 or C^∗^04:01.

**Table 2:** Peptides derived from the HLA molecule itself identified

Sequence	Protein	Start	End	Gene	Intensity											
					C1R			B∗14:02			B∗14:03			B∗27:05		
					1A	1B	1C	2A	2B	2C	3A	3B	3C	4A	4B	4C
RRYLENGK	Q04826	193	200	HLA	0	0	0	0	0	0	2,6E+07	5,1E+07	1,8E+07	7,4E+08	1,1E+09	4,9E+08
RRYLENGKE	Q04826	193	201	HLA	0	0	0	0	0	0	6,8E+07	1,0E+08	3,4E+07	2,1E+09	2,6E+08	1,0E+08
RRYLENGKET	Q04826	193	202	HLA	0	0	0	0	0	0	4,6E+08	6,5E+08	2,3E+08	3,0E+08	6,2E+08	2,2E+08
RRYLENGKETL	Q04826	193	203	HLA	0	0	0	0	0	0	1,7E+08	2,5E+09	2,3E+09	2,3E+10	2,4E+10	2,3E+10
RRYLENGKETLQ	Q04826	193	204	HLA	0	0	0	0	0	0	0	0	0	1,3E+09	1,8E+09	9,2E+08
RYLENGKETL	Q04826	194	203	HLA	0	0	0	0	0	0	0	0	0	4,1E+07	2,4E+07	1,1E+07
LENGKETL	Q04826	196	203	HLA	0	0	0	0	0	0	0	0	0	0	4,6E+06	0

The table displays the peptides derived from the HLA molecule itself, identified as bound to HLA-B∗14 and HLA-B∗27:05 in this study.

HLA, human leukocyte antigen.

Collectively, this data could suggest a potential role for HLA-derived self-peptides in disease susceptibility, aligning with previous studies (29, 30, 31, 32)

### Overlap Between B∗14 and B∗27 Subtypes

**Fig. 9:**
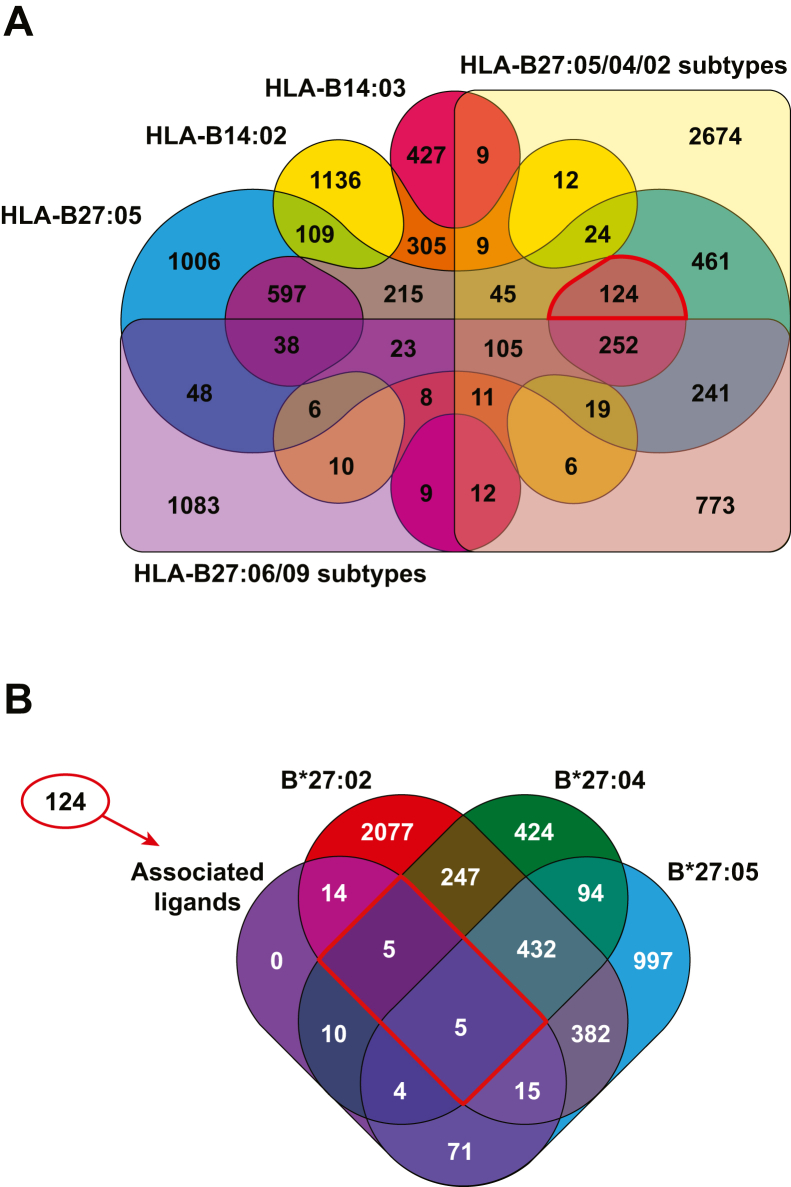
**Overlap between subtypes of B∗14 and B∗27**. *A*, Venn diagram with the HLA-B∗27:05, -B∗14:02, and -B∗14:03 ligands identified in this study and with those of the B∗27 subtypes previously described in (33). *B*, Venn diagram comparing the 124 peptides exclusively identified in the AS-associated subtypes with the peptides previously identified in the HLA-B∗27 subtypes 02, 04 and 05. AS, ankylosing spondylitis; HLA, human leukocyte antigen.

If susceptibility to AS was indeed dependent on the recognition of specific peptides bound to different arthritogenic HLA-I molecules, one would expect to find shared ligands between B∗27:05 and B∗14:03 capable of binding to other AS-associated HLA-B∗27 subtypes and which would not be presented by non-associated subtypes of B∗27 or by B∗14:02. To test whether this was the case, we compared the B∗27:05, B∗14:02, and B∗14:03 ligandomes identified in this work with those of the B∗27 subtypes most commonly associated with AS (HLA-B∗27:02, B∗27:04 and B∗27:05) and with those not associated (B∗27:06 and B∗27:09) previously identified in the same cell line (33). Of the 1011 ligands that we found to be common in the B∗27:05 and B∗14:03 peptidomes and absent from that of B∗14:02, 597 had not been previously reported as binding to the major B∗27 subtypes in the study by Schittenhelm *et al*. and may exhibit differential binding to these B∗27 subtypes. Among the 414 previously identified ligands, 124 were found exclusively in one or more of the associated B∗27 subtypes (Table S2). Notably, within this subset, 10 ligands (ARVSIVNQY, KRNPGVKEGY, ARLAQRIDF, GRMDGSLGL, QRKFPHLEF, GRSDLIPTI, RRFAPDISSY, LRHYGYLRF, GRNKFGQLGL, and ARSLQKLGF) were previously identified bound to both B∗27:02 and B∗27:04 and were not found among the ligands of the non-associated subtypes of B∗27 (Fig. 9, Table 3) (33). The only statistically significant difference between these 10 ligands and the other 1001 is the presence of aromatic residues at the Ct position (7/10 *versus* 226/1001, *p* = 0.008).

**Table 3:** Peptides identified in HLA-B∗27:05 and HLA-B14:03 overlapping with HLA-B∗27:02 and -B∗27:04 and absent in HLA-B∗14:02, -B∗27:06, and -B∗27:09

Sequence	Length	Protein	Start position	End position	Protein names
ARLAQRIDF	9	A0A1W2PRX4	41	49	Mediator of RNA polymerase II transcription subunit 17
ARSLQKLGF	9	Q7Z6U1	85	93	TATA box-binding protein-like protein 1
ARVSIVNQY	9	A0A0C4DG22	170	178	RNA exonuclease 4
GRMDGSLGL	9	Q15751	3553	3561	Probable E3 ubiquitin-protein ligase HERC1
GRNKFGQLGL	10	H0Y6K7	212	221	Probable E3 ubiquitin-protein ligase HERC4
GRSDLIPTIKF	11	Q14691	54	64	DNA replication complex GINS protein PSF1
KRNPGVKEGY	10	D6RF62	18	27	Multifunctional protein ADE2
LRHYGYLRF	9	Q15036	266	274	Sorting nexin-17
QRKFPHLEF	9	F5GY90	138	146	Porphobilinogen deaminase
RRFAPDISSY	10	G5E9V4	130	139	Hermansky-Pudlak syndrome 3 protein

The table shows the peptides identified in this study bound to both HLA-B∗27:05 and HLA-B∗14:03, which overlap with peptides previously identified in the HLA-B∗27 subtypes 02 and 04 (33) and are absent in HLA-B∗14:02, -B∗27:06, and -B-27:09.

HLA, human leukocyte antigen.

Of the four peptides derived from the HLA molecule itself and presented by the associated subtypes B∗14:03 and B∗27:05 (Table 2), the longest peptide, RRYLENGKETL, was also identified in the study by Schittenhelm *et al*. (33) bounds to HLA-B∗27 subtypes not associated with the disease. However, the other three peptides were not identified in that study.

The existence of shared ligands between the compared AS-associated allotypes (HLA-B∗27:02, B∗27:04, B∗27:05, and B∗14:03) opens the possibility that one or more peptides could specifically bind to the AS-associated subtypes of B∗27 and B∗14:03 and act as arthritogenic self-peptides.

## Discussion

Recent findings reveal that peptide-HLA-I molecules are highly dynamic, adjusting their conformation in response to minor HLA-I polymorphisms or peptide interactions, with significant biological implications and deepen our insight into the molecular mechanisms that regulate T cell receptor (TCR)-mediated antigen discrimination (34). HLA alleles that differ by only a single amino acid can have distinct disease associations due to subtle yet crucial effects on antigen presentation and immune recognition (35). This is the case for B∗14:02 and B∗14:03, which differ only at residue 156 and are differentially associated with AS. Such differences can alter the conformation of the peptide-binding groove, affecting peptide selection, stability, or interactions with the TCR. Even minor structural differences between B∗27:05 and B∗27:09 can influence TCR recognition, potentially modifying immune responses and triggering autoimmunity (36). These findings highlight how single-residue variations in HLA molecules can dramatically alter disease risk by modulating antigen presentation, TCR engagement, or even interactions with other immune receptors like NK cell inhibitory receptors.

In our present study, we have assessed and compared the ligandomes of B∗14:02, B∗14:03, and B∗27:05 with up-to-date MS sequencing technology. We found 1011 peptides common between the two AS-associated alleles and not present in B∗14:02, which supports the presentation of common arthritogenic peptides by different AS-associated HLA-I molecules as the basic mechanism for AS. The overlap in peptide repertoires between HLA-B∗27:05 and HLA-B∗14:03, despite their structural differences, likely results from a combination of factors related to the architecture and biochemical properties of their binding grooves. We propose that similarities in the B and F pockets may allow both molecules to accommodate overlapping peptide motifs, even if other regions of the protein differ. Additionally, conformational plasticity and secondary anchor residues may contribute to peptide compatibility. Finally, electrostatic similarities in the binding grooves of these molecules could further stabilize interactions with peptides carrying specific motifs. In the absence of new experimental data, we speculate that these structural and biochemical factors may help explain the observed repertoire overlap beyond simple sequence similarity.

One of the main properties that characterize the peptide repertoire of B∗27 is the presence of R at P2, something uncommon in the rest of HLA molecules except for B∗14 and B∗39. HLA-B∗39 can bind some HLA-B∗27 ligands (37) and is also associated with AS in HLA-B∗27 negative individuals (38). Different HLA alleles capable of binding peptides with arginine at P2 as an anchor motif differ by up to seven amino acids in the P2 environment. These variations must therefore preserve the interaction between the HLA molecule's B pocket and the arginine at P2 of the peptide (39). These data, together with our results, are compatible with the existence of one or more peptides capable of binding to HLA-B∗27, -B∗39, and -B∗14:03 that could have a role in AS.

Our findings align with previously published HLA-B∗27:05 ligandomes, consisting mainly of 9- and 10-mers peptides with a primary anchor position of R at P2, a secondary anchor position of basic, aliphatic, or aromatic residues at the Ct and P1 with a strong preference for G and R (40, 41). However, we also detected an enrichment in residues K, A, and S in the latter position.

The motifs of B∗14:02 ligands were also analyzed by DiBrino *et al*. (42). Like us, they described an enrichment in acidic residues at P1, R at P2 and P5, aliphatic/aromatic residues at P3, and L at C terminus, but they also found an enrichment in I and L at P6 that we did not find. Unlike that report, we also identified a significant number of the B∗14:02 peptides with Q, P or E at P2, whose majority (80%) contained R in P5, which could be an alternative anchor motif in the absence of R at P2. The previous study observed that, although R occurred at high frequency at P5 or P6 in endogenous peptides, its substitution in these positions in stabilization assays did not prevent binding to B∗14:02. However, those stabilization assays used synthetic peptides that always contained R at P2. Thus, it is still possible that the anchor motif at P5 has a relevant role in the absence of R at P2.

Only 28 HLA-B∗14:03 ligands were identified in previous studies (18), and thus the anchor motifs of this molecule were not accurately defined. In our work, we observed that most of the ligands of this molecule present R at P2, aliphatic or aromatic residues at the Ct, R/K/A/G/S at P1, and a low proportion of R at P5. Thus, the distribution of residues is very similar at positions P1, P2, and P5 between the two alleles associated with AS, while at the Ct position the HLA-B∗14:03 ligands are more similar to the ones of B∗14:02. This was surprising, since while there are more than 20 differences between the amino acidic sequences of B∗14:03 and B∗27:05, the majority of which in the α1 and α2 domains, the 2 B∗14 subtypes differ only at position 156 (B∗14:02^156^L, B∗14:03^156^R), which is part of the D and E pockets and, therefore, expected to have a direct influence only on the central region of the bound peptides. However, residue 156 could have long-range indirect effect on residues close to the A pocket that could modulate P1 and P2 residue preferences. Some studies have proposed that a basic residue at position 156 (mainly R, as in B∗14:03) could directly interact with the P5 position of the peptide (43, 44). Since R at P5 in B∗14:02 seems to be a secondary anchor position, the change in B∗14:03^156^R could cause a charge change that would disrupt the interaction with R at P5, indirectly modulating the preference for certain residues in distant positions such as P1 and P2. In the previous crystallography studies, in the crystal structures of HLA-B∗14:02 and HLA-B∗27:05 with two different peptides, only one of the two peptides adopted a similar conformation in the two molecules (17). Notably, the viral peptide pLMP2, which adopted a different conformation, contained an R residue at position P5. In our virtual L156R mutation in the crystal structure of HLA-B∗14:02 with the viral peptide pLMP2 mainly affected the interaction between the residue 156 with P3 of the peptide, as expected, but also led to the interaction between residue ^156^R with W97, Y99, and Y159 of B∗14 molecule, which in turn interacted with positions P1, P2, P3, and P5 of the peptide. Interestingly, a cationic interaction between W97 and P5 that was observed in B∗14:02 could disappear from B∗14:03, which could explain the absence of the secondary anchor motif in B∗14:03. Interestingly, the polymorphism at position 156 also affects the interaction with the peptide loading chaperone tapasin (45) and alter cytotoxic T lymphocyte (CTL) recognition (46, 47). However, the interaction with tapasin was stronger for B∗27:05 than for B∗14:02 and stronger for B∗14:02 than for B∗14:03, suggesting that the peptide repertoires of B∗14:02, and especially B∗14:03, were less optimized than that of B∗27:05 (13).

We identified 1011 peptides common to the peptidomes of the AS-associated alleles and absent from the peptidome of non-AS–associated allele while in the previous study none were detected in that subgroup (18). The greatest difference of these peptides compared with the other subgroups resides in the amino-terminal residue usage, where almost 70% of the 1011 peptides had R, K, A, or G. A significant difference between the repertoires of B∗27:05 and B∗14:03 was found at the Ct position: while B∗27:05 bound peptides with greater diversity of amino acids at this position (basic, aliphatic, or aromatic residues), the repertoire of B∗14:03 was mainly restricted to aliphatic residues (mainly L) and F. Consequently, the common repertoire between these two molecules presented a restriction similar to that of B∗14:03 at the Ct, although with a significant increase in the proportion of F and a decrease in the proportion of L. The nature of the accepted Ct residues might be important, since initial studies suggested that the non-AS–associated allotypes B∗27:06 and B∗27:09 did not bind peptides with Y at the Ct, a residue that is accepted by disease-associated subtypes B∗27:05, B∗27:02, and B∗27:04, thus suggesting that putative arthritogenic peptides could carry this anchor motif (48, 49). However, none or few ligands with Y at Ct were observed for the AS-associated B∗27:07 allele (33, 50) and for our B∗14 subtypes. Thus, it seems unlikely that common peptide among the disease-associated subtypes would have to contain Y at the Ct position.

Importantly, we identified 10 peptides shared between the AS-associated subtypes B∗27:05 and B∗14:03 in our study, as well as with the strongly AS-associated subtypes B∗27:02 and B∗27:04 in (33) and absent in all the studied non-associated alleles, B∗14:02 in our study and B∗27:06 and B∗27:09 reported in (33). Schittenhelm *et al*. did not find any peptide ligand capable of binding to all disease-associated B∗27 subtypes that they studied, including the low frequency alleles B∗27:03, B∗27:07, and B∗27:08 and absent in the non-associated ones, which led them to study whether quantitative changes in ligand abundance between the different B∗27 subtypes could be relevant for activation of self-reactive T cells (28, 33). They identified 26 ligands that were more abundant in the HLA-B∗27 subtypes associated with AS than in the two non-associated ones, and they considered them candidates of arthritogenic peptides (28). In our study, we identified eight of these peptides. Two of them were also found in the HLA-B∗14:02 ligandome, five of them were exclusively bound to HLA-B∗27:05 and, one of them, the TRYDLYHTF peptide, was identified exclusively bound to B∗27:05 and B∗14:03. Interestingly, this peptide has a high homology with different bacterial sequences, being the highest homology with the regions 298 to 306 (TRYDLYRTF) of the zeta toxin family protein from Rhodococcus, a bacterial genus altered in the microbiota of patients with autoimmune diseases such as rheumatoid arthritis (51) and inflammatory bowel disease (52), pointing to that peptide as another candidate for arthritogenic peptide.

The presence of self-peptides derived from HLA molecules themselves represents an alternative factor in distinguishing disease-associated HLA subtypes. Previous studies have suggested that HLA-B∗27-restricted peptides, particularly those from the 169 to 181 regions, could contribute to autoimmune diseases by acting as molecular mimics or by inducing aberrant immune responses (29, 30, 31, 32). Our data confirm that peptides from this region are selectively presented by disease-associated HLA-B alleles, aligning with previous findings on the role of HLA-B∗27–derived peptides in autoimmunity. The peptides RRYLENGK, RRYLENGKE, RRYLENGKET, and RRYLENGKETL, in particular, were bound exclusively to disease-associated subtypes (HLA-B∗27:05 and HLA-B∗14:03) while absent in HLA-B∗14:02, a non-disease-associated allele. The immunological relevance and potential role of these peptides in autoimmune pathogenesis of AS requires further investigation.

Recently, the isolation of orphan TCRs with a disease-associated BV9–CDR3β motif from AS patients, combined with HLA-B∗27:05 peptide library screening, identified shared self and microbial peptides capable of activating AS TCRs. Structural analysis revealed a common binding motif in TCR cross-reactivity for peptide-MHC, supporting the role of microbial and self-antigens in HLA-B∗27–associated disease (53). Therefore, based on our observations, the association of B∗14:03 subtype with AS seems to be compatible with the specific presentation of one or more peptides, which could also be presented by the B∗27 subtypes associated with AS. However, we acknowledge that peptidome comparisons alone may not be sufficient to fully explain AS pathogenesis, and further functional studies are needed to clarify the role of these peptides in disease development. The possibility that some alloreactive CTL clones raised against HLA-B∗14:02 can recognize HLA-B∗27:05 suggests that T cell cross-reactivity between these allomorphs is not implausible. Notably, B∗14:02 and B∗27:05 differ by one additional amino acid compared to the difference between B∗14:03 and B∗27:05, yet cross-reactive CTLs were still detected, albeit at a low frequency (2.9%) (18). This raises the possibility that some TCR clonotypes could recognize both B∗14:03 and B∗27:05, particularly given their peptide repertoire overlap. However, cross-reactivity could arise through multiple mechanisms. One possibility is that the same peptide, when bound to each molecule, engages distinct TCR clonotypes, leading to different immune responses. Alternatively, it cannot be ruled out that entirely different peptides presented by B∗14:03 and B∗27:05 contribute to disease pathogenesis. While this scenario is conceivable, the significant overlap in the peptide repertoires of B∗27 and B∗14:03 makes the hypothesis of a single shared arthritogenic peptide more compelling. Ultimately, further studies on TCR specificity and peptide presentation are needed to clarify whether a common ligand is truly responsible for the observed clinical associations.

In conclusion, our study sheds light on the intricate peptide binding characteristics of HLA-B∗27:05 and HLA-B∗14:03, both strongly associated with AS, in contrast to the non-AS–associated HLA-B∗14:02. Our findings reveal a remarkable overlap in peptide repertoires between B∗14:03 and B∗27:05, despite their significant structural differences, while B∗14:02 shows distinct binding preferences. The identification of 1011 shared ligands between B∗14:03 and B∗27:05, absent in B∗14:02, suggests a potential mechanism underlying AS development through common peptide recognition. Moreover, our modeling suggests that a single residue variation at position 156 in B∗14 alleles may induce long-range effects on peptide binding, influencing the distribution of specific amino acids at crucial positions.

## Data Availability

The mass spectrometry proteomics data have been deposited to the MassIVE repository (http://massive.ucsd.edu) with the dataset identifier MSV000095860 (ftp://MSV000095860@massive.ucsd.edu, password: EL_2024). The list of peptides identified is shown in Supplemental Table S1.

## Supplemental Data

This article contains supplemental data.

## Conflict of Interest

The authors declare no competing interests.

## Figures and Tables

**Supplemental Figure S13:**
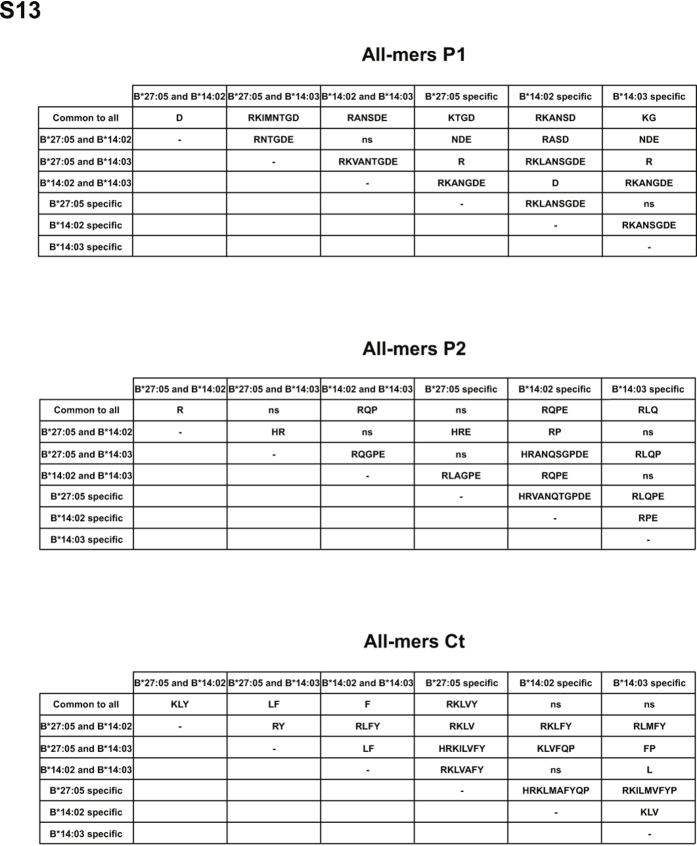

